# Optimized production of transgenic buffalo embryos and offspring by cytoplasmic zygote injection

**DOI:** 10.1186/s40104-015-0044-x

**Published:** 2015-10-20

**Authors:** Fanli Meng, Hui Li, Xiaoli Wang, Guangsheng Qin, Björn Oback, Deshun Shi

**Affiliations:** Animal Reproduction Institute, State Key Laboratory for Conservation and Utilization of Subtropical Agro-bioresources, Guangxi University, 75 Xiuling Road, Nanning, 530005 P.R China; Present address: AgResearch Ltd., Ruakura Research Centre, Reproductive Technologies, 10 Bisley Road, Private Bag 3123, Hamilton, New Zealand

**Keywords:** Buffalo, Cytoplasmic injection, Plasmid, Transgenic, Zygote

## Abstract

**Background:**

Cytoplasmic injection of exogenous DNA into zygotes is a promising technique to generate transgenic livestock. However, it is still relatively inefficient and has not yet been demonstrated to work in buffalo. We sought to improve two key technical parameters of the procedure, namely i) how much linear DNA to inject and ii) when to inject it. For this, we introduced a constitutively expressed enhanced green fluorescent protein (EGFP) plasmid into buffalo zygotes.

**Results:**

First, we found that the proportion of EGFP-expressing blastocysts derived from zygotes injected with 20 or 50 ng/μL DNA was significantly higher than from those injected with 5 μg/mL. However, 50 ng/μL exogenous DNA compromised blastocyst development compared to non-injected IVF controls. Therefore the highest net yield of EGFP-positive blastocysts was achieved at 20 ng/μL DNA. Second, zygotes injected early (7–8 h post-insemination [hpi]) developed better than those injected at mid (12–13 hpi) or late (18–19 hpi) time points. Blastocysts derived from early injections were also more frequently EGFP-positive. As a consequence, the net yield of EGFP-expressing blastocysts was more than doubled using early vs late injections (16.4 % vs 7.7 %). With respect to blastocyst quality, we found no significant difference in cell numbers of EGFP-positive blastocysts vs non-injected blastocysts. Following embryo transfer of six EGFP-positive blastocysts into four recipient animals, two viable buffalo calves were born. Biopsied ear tissues from both buffalo calves were analyzed for transgene presence and expression by Southern blot, PCR and confocal laser scanning microscopy, respectively. This confirmed that both calves were transgenic.

**Conclusions:**

Our cytoplasmic injection protocol improved generation of transgenic embryos and resulted in the first transgenic buffalo calves produced by this method.

## Background

Livestock transgenesis is a tool for elucidating gene function and can also play a key role in many biotechnological applications, such as establishing genetic disease models and producing new animal products [1]. In the past, transgenic farm animals have been generated by pronuclear microinjection (reviewed in [2]), somatic nuclear transfer cloning (SCNT) and lentiviral infection (reviewed in [3]). Gene transfer by pronuclear microinjection has a low success rate for generating transgenic livestock with 100 % germline transmission [4, 5]. It has been reported that transgene expression in bovine and porcine embryos is only approximately 3 % and 20 %, respectively, with the majority of embryos being mosaics [6, 7]. Overall, only about 1 % of livestock embryos from pronuclear injection develop into transgenic founders, posing a major obstacle in transgenic animal production. Likewise, SCNT only results in about 1–5 % of transgenic embryos developing into fertile live offspring, mainly owing to genetic and epigenetic abnormalities associated with the cloning procedure [8]. Lentiviral infection of early embryos has increased the efficiency of transgenesis to 10–30 % [9]. However, these vectors have other drawbacks, including i) restricted transgene size, ii) silencing of viral DNA, and iii) creation of mosaic animals, in which only some cells carry the transgene [10].

Cytoplasmic DNA injection has emerged as an alternative method to introduce foreign genes into zygotes. Compared to traditional pronuclear injection, this technique does not require visualization of the male and female pronuclei. This is an advantage for zygotes whose high lipid contents disguise the pronuclei, such as in cattle, sheep and pig [11]. In these species, zygotes are centrifuged to visualize the pronuclei, an approach which may compromise their developmental capacity [5]. Using cytoplasmic injection, transgenic mice [12–15], rats [16], cynomolgus monkey [17], cattle [18] and pigs [9–21] have been produced. Using condensed DNA in combination with electroporation, 2.4 % of injected mouse zygotes developed into transgene-expressing blastocysts [13]. In order to further increase efficiency, high concentrations (625 ng/μL) of divalent cation-complexed DNA was injected, resulting in a net yield of up to 7.4 % of EGFP-positive murine morulae/blastocysts [13]. In cattle, injection of naked DNA into IVF zygotes did first not yield EGFP-positive blastocysts, however, by injecting DNA–liposome complexes the rate increased to 12 % [22].

In the present study, we introduced linear DNA into buffalo zygotes by cytoplasmic injection. By varying the amount of exogenous DNA and time point of injection, we achieved a doubling in the net production of transgene-expressing blastocysts. Following blastocyst transfer into surrogate recipients, we obtained viable transgenic buffalo calves. Taken together, we established a robust technique that resulted in the first transgenic buffalo using an optimized cytoplasmic injection protocol.

## Methods

### Reagents and media

Chemicals were purchased from Sigma-Aldrich (St. Louis, MO, USA) and all embryo manipulations were carried out on a warm stage (38.5 °C), unless indicated otherwise.

### DNA preparation

The transgenic cassette, a 4.7 kb pEGFP-N1 plasmid encoding the EGFP gene driven by the CMV promoter (GenBank Accession #U55762, Clontech, USA), was purified by using an endotoxin-free kit (QIAGEN, USA), according to the manufacturer’s instructions. The purified plasmid was digested by *Apa*L I at position 4361, resulting in linear molecules with similar staggered (“sticky”) ends, and the gel extract purified by using the QIAEX II Extraction Kit. DNA concentration was determined by using a NanoDrop™ 1000 spectrophotometer (Thermo Fisher Scientific Inc., Waltham, MA, USA). Linearized DNA was diluted to 100 ng/μL in MilliQ® water, aliquoted and stored at −20 °C until use.

### In vitro maturation (IVM) and fertilization (IVF) of embryos

Water buffalo ovaries (*Bubalus bubalis*) were collected from a local abattoir within 20–30 min after slaughter and transported to the laboratory in a thermos containing phosphate-buffered saline (PBS) at 30–35 °C within 4–6 h. Buffalo cumulus-oocyte complexes (COCs) were recovered by aspiration of buffalo follicles (diameter 2–6 mm) using a 10 mL disposable syringe with 18-gauge needle. Only oocytes with compact, non-atretic cumulus oophorus-corona radiata, and a homogenous ooplasm were selected for IVM. The IVM medium comprised TCM-199, supplemented with 26.2 mmol/L NaHCO_3_, 5 mmol/L HEPES, 5 % estrous cow serum (OCS, self-preparation), 2 % bovine follicular fluid (BFF) and 0.1 ng/μL FSH). COCs were transferred to a 35 mm glass dish containing 1.5 mL IVM medium and cultured for 20–22 h under a humidified atmosphere of 5 % CO_2_ in air at 38.5 °C. After IVM, buffalo COCs were fertilized with proven water buffalo sperm using our standard IVF procedure [23].

### Cytoplasmic injection

Presumptive buffalo zygotes which had extruded the second polar body were selected and transferred to a 50 μL drop of culture medium in a 60 mm dish overlaid by mineral oil. pEGFP-N1 plasmid was loaded into a microinjection needle (inner tip diameter 4–5 μm). Using manual micromanipulators (NT88-V3, Narishige, Japan) and micro-injectors (CellTram® Oil, Eppendorf, Germany) mounted to an inverted microscope (Nikon T300, Japan), approximately 12 pl plasmid was injected into the zygote cytoplasm. The estimated injected volume (V) was calculated from the average inner radius of the injection needle (*r* = 4.5 μm) and length of the injected liquid column (h = 200 μm) by using the formula V = πr^2^ * h. The basic micromanipulation medium was TCM-199, supplemented with 5 mmol/L NaHCO_3_, 5 mmol/L HEPES and 5 % OCS. After injection, the zygotes were washed twice in TCM-199, supplemented with 3 % OCS culture medium and transferred into culture drops.

### In vitro culture (IVC)

Following cytoplasmic injection, 10–15 zygotes were transferred to a 30 μL drop of culture medium and co-cultured with primary cumulus cells. All cultures were overlaid with mineral oil and done in an incubator with 5 % CO_2_ in humidified air at 38.5 °C. The culture medium was replaced every 48 h. Cleavage was evaluated 48 h after IVC, and the number of morulae and blastocysts determined on D7.

### EGFP expression in pre-implantation embryos

EGFP expression was observed under an epifluorescence inverted microscope (NikonT300, Japan) on D7. Briefly, different stages of implantation embryos were exposed to blue light (excitation wavelength 488 nm, emission wavelength 530 nm), the EGFP expression signal was observed and fluorescent photos acquired with a CCD camera (DS-5Mc, Nikon, Japan).

### Determination of embryo cell numbers

Embryos were stained with 1 mg/mL Hoechst 33342 (B2261) for 10 min, washed twice in PBS and single blastocysts mounted into a drop of acid solution (50 mL MilliQ® H_2_O + 100 μL 5 N HCl + 50 μL Tween-20) on glass slides. Images were acquired as described above and total nuclei numbers were manually counted.

### Embryo transfer and pregnancy monitoring

Embryo development into blastocysts was assessed seven days after insemination (D7). EGFP-positive blastocysts were identified using an inverted fluorescence microscope as described above. EGFP-positive blastocysts were scored, and morphological grade 1 and 2 blastocysts (i.e. with a symmetrical and spherical ICM of uniform size, color and density) were selected for embryo transfer. Recipient hybrid buffalo, derived from crossing local swamp buffalo breeds with Murrah river buffalos and purchased from local farmers, were synchronized as described [24]. On D7 following estrus (estrus = D0 = day of IVF), EGFP-positive blastocysts were transferred non-surgically into the uterine lumen ipsilateral to the corpus luteum. The pregnancy status of recipient cows was determined on D40 of gestation by using ultrasonography (Aloka SSD-500 scanner with a 5 MHz linear rectal probe, Aloka Co Ltd, Tokyo, Japan).

### Transgene detection

Buffalo calves were delivered after gestation. To detect the integration of exogenous genes in these transgenic buffalo calves, genomic DNA from ear tissue was extracted and used as template to set up polymerase chain reaction (PCR). EGFP- specific amplification was performed by using the following primer pair: Forward: 5'- CTGGTCGAGCTGGACGGCGAC (724–744 in pEGFP-N1: within EGFP coding sequence) -3'; Reverse: 5'-CTACAAATGTGGTATGGCTGA- (1443–1423 in pEGFP-N1: between EGFP coding sequence and SV40 poly A) 3′; PCR conditions were: pre-denature 95 °C for 5 min, and then 95 °C for 45 s, 60 °C for 45 s, 72 °C for 45 s for 35 cycles, and a last extension at 72 °C for 7 min; the PCR product size was 720 bp.

Integration of the EGFP gene was also determined by Southern blot. Briefly, 20 μg of genomic DNA from ear tissue was digested with *Bam*H I*,* separated on a 1 % agarose gel and blotted onto a nylon membrane. An EGFP probe fragment was amplified by using the same primers as for PCR. Genomic DNA extracted from non-transgenic buffalo ear tissue was used as a negative control. Random-primed DIG-11-dUTP labeling of the DNA probe was carried out using the ‘High Prime DNA Labeling and Detection Starter Kit II’ (Roche, USA), according to the manufacturer’s instructions.

### Detection of EGFP expression

Buffalo ear tissue was harvested by removing the hairs with a scalpel blade, rinsing thoroughly in cold PBS, and fixing the cells in 4 % fresh paraformaldehyde at 4 °C overnight. The tissues were washed and perfused in gradient concentration of sucrose solution (5 %, 10 %, 15 %, 30 %) at 4 °C, before embedding in CRYO-OCT Tissue-Tek™ (Fisher Scientific, USA). Cryo-sections were cut at 15 μm thickness and observed under a confocal laser scanning microscope (Zeiss LSM 510META, Germany) to identify EGFP expression. Images were acquired with an AxioCam (Zeiss), keeping all microscope and laser settings kept constant between different groups and replicates. Brightfield and fluorescent images were digitally enhanced for brightness and contrast in Corel Paint Shop Pro XI (‘Histogram adjustment’). The same settings were used for images of all three groups.

### Statistical analysis

Statistical significance was accepted at *P* < 0.05 and determined using the two-tailed Fisher exact test for independence in 2 x 2 tables for developmental data and transgene expression (Tables 1, 2) or the paired two-tailed Student *t*-test for cell counts. All values are presented as mean ± SD, unless indicated otherwise.

**Table 1:** Effect of injected DNA concentration on embryo development and transgene expression

DNA Concentration	n	N	Cleavage(% ± SD)	Blastocyst development	EGFP^+^ Blastocysts	Net yield EGFP^+^
				(% ± SD)^a^	(% ± SD)^b^	Blastocysts (% ± SD)^c^
0 ng/μL	3	57	49 (86.0 ± 4.9)	18 (31.6 ± 4.1)^d^	0	0
5 ng/μL	3	110	83 (75.5 ± 2.1)	28 (25.5 ± 1.7)^de^	8 (28.6 ± 5.6)^d^	7.3 ± 1.6
20 ng/μL	3	98	72 (73.5 ± 1.8)	22 (22.4 ± 6.8)^de^	15 (68.2 ± 5.8)^e^	15.3 ± 3.7
50 ng/μL	3	106	76 (71.7 ± 5.6)	15 (14.2 ± 3.8)^e^	12 (80.0 ± 18.0)^e^	11.3 ± 3.8

*n* number of independent experiments;^a^ proportion of embryos placed into IVC (N) that developed into D7 blastocysts (B) grade 1–3;^b^ proportion of EGFP^+^ blastocysts out of total D7 blastocysts;^c^ proportion of EGFP^+^ blastocysts out of N;^d, e^ rows with different superscripts within a column differ *P* < 0.05 from non-injected control (‘0 ng/μL’)

**Table 2:** Effect of injection timing on embryo development and transgene expression

Time point of injection	n	N	Cleavage (% ± SD)	Blastocyst development	EGFP^+^ Blastocysts	Net yield EGFP^+^
				(% ± SD)^a^	(% ± SD)^b^	Blastocysts (% ± SD)^c^
No injection	3	71	59 (83.1 ± 2.6)	25 (35.2 ± 5.5)	0	0
7–8 hpi	3	116	100 (86.2 ± 3.6)	30 (25.9 ± 3.9)	19 (63.3 ± 5.9)^d^	16.4 ± 5.9
12–13 hpi	3	102	83 (81.4 ± 3.8)	23 (22.5 ± 1.4)	12 (52.2 ± 2.9)^d, e^	11.8 ± 2.9
18–19 hpi	3	78	62 (79.5 ± 5.5)	16 (20.5 ± 9.1)	6 (37.5 ± 2.9)^e^	7.7 ± 2.9

*n* number of independent experiments;^a^ proportion of embryos placed into IVC (N) that developed into D7 blastocysts (B) grade 1–3;^b^ proportion of EGFP^+^ blastocysts out of total D7 blastocysts;^c^ proportion of EGFP^+^ blastocysts out of N;^d, e^ rows with different superscripts within a column differ *P* < 0.05 from non-injected control

**Fig. 1:**
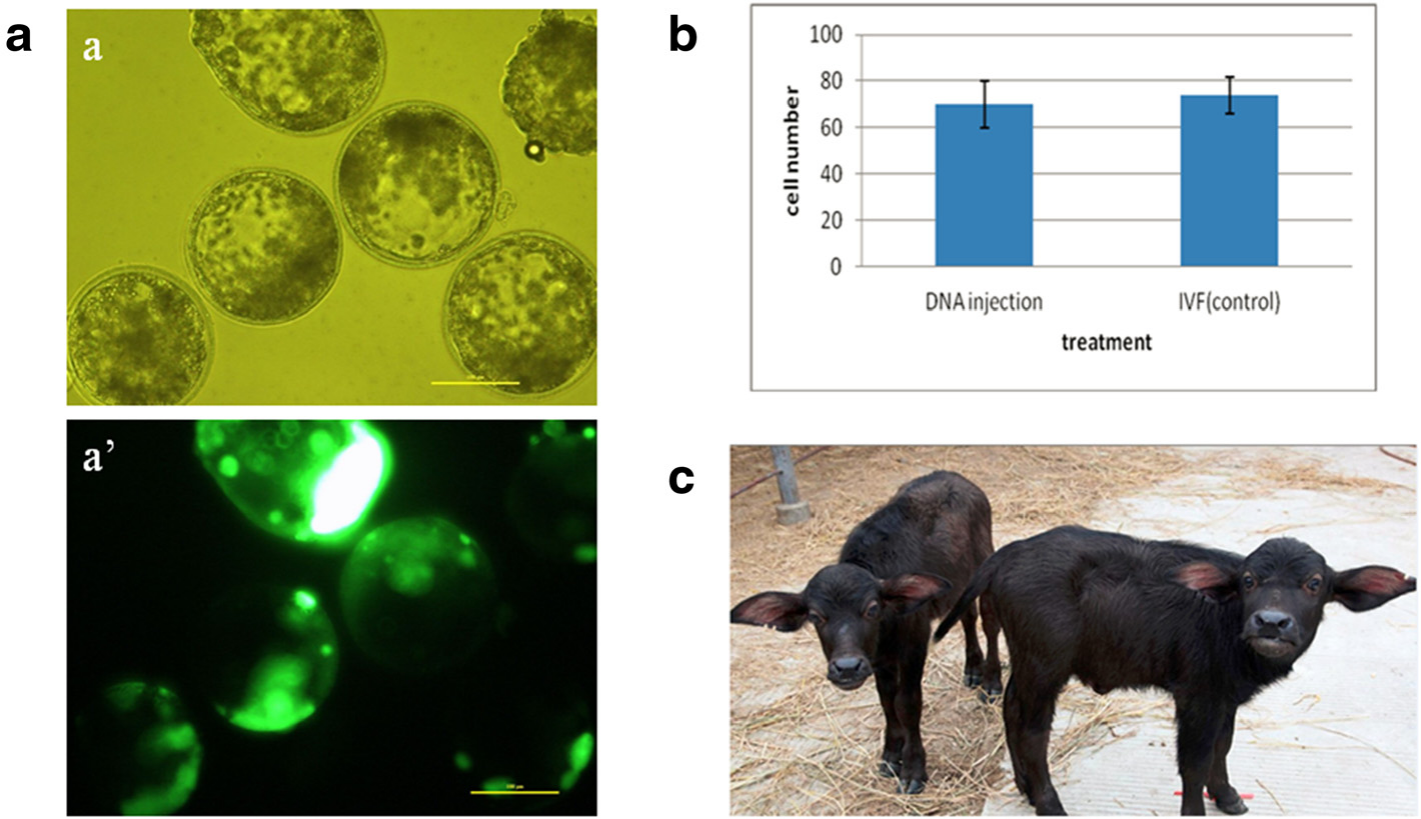
Characterization of EGFP-positive buffalo blastocysts. **a**. Microscopic evaluation by brightfield (a) and wide-field epifluorescence (a’); scale bar =100 μm. **b**. Nuclei numbers in EGFP-positive and non-injected IVF blastocysts. **c**. Mixed-sex twin buffalo calves born in December 2010

## Results and discussion

We focused on two parameters for optimization, namely i) DNA concentration and ii) injection time point.

### Optimizing amount of injected DNA

In principle, higher amounts of exogenous DNA should favor DNA integration into the genome. Accordingly, we observed an increase in EGFP-positive blastocysts with increasing DNA concentrations. For the dose–response test, 5, 20 and 50 ng/μL exogenous linear DNA (corresponding to ~1x10^4^, ~4x10^4^ and ~1x10^5^ copies, respectively) were introduced into buffalo zygotes 7–8 h after IVF. For a similar size plasmid, this represents ~100-fold more injected DNA molecules than in a comparable mouse study [14]. Following seven days of IVC, embryo development and EGFP expression were determined (Table 1, Fig. 1a). The rate of EGFP-positive blastocysts was significantly higher in both 20 ng/μL and 50 ng/μL compared to 5 ng/μL (68.2 ± 5.8 % or 80.0 ± 18.0 % *vs.* 28.6 ± 5.6 %, *P* < 0.05). These findings are comparable to the 44–71 % fluorescent blastocysts previously achieved by injecting 10 ng/μL plasmid into bovine zygotes [25]. However, embryo development was compromised at 50 ng/μL compared to non-injected IVF controls (14.2 ± 3.8 % *vs.* 31.6 ± 4.1 %, *P* < 0.05). A similar inverse relationship between efficiency of transgenesis and embryo development was previously observed in mouse pronuclear [26] and cytoplasmic injection [12]. We therefore used 20 ng/μL DNA for optimizing the injection time point in the next set of experiments.

### Optimizing time point of injection

For efficient transgene integration, the embryonic genome has to be accessible to the exogenous DNA. Therefore, the timing of DNA injection with respect to the period of pronuclear formation and chromatin remodeling is critical. We next compared different time points of injection with respect to embryo development and frequency of transgene expression (Table 2). Buffalo zygotes were cytoplasmically injected at 7–8 hpi, 12–13 hpi and 18–19 hpi. In analogy to bovine, these time points should roughly correspond to early (pronuclei stages PN1-3), medium (PN3-6) and late (PN5-6) pronuclei formation, respectively [27]. Non-injected IVF zygotes served as control. There were no significant differences in cleavage rates between these time points. Blastocyst development tended to be better at earlier time points but these differences were not significant. Accordingly, the proportion of EGFP-positive blastocysts was significantly higher at 7–8 hpi vs 18–19 hpi (63.3 ± 5.9 % vs 37.5 ± 2.9 %, *P* < 0.05) and their net yield was more than doubled between these two time points (16.4 ± 5.9 % vs 7.7 ± 2.9 %, *P* = 0.12). A similar increase in EGFP-positive blastocysts was previously observed when DNA-liposome complexes were injected into bovine oocytes vs zygotes at 16 hpi, resulting in net efficiencies of 0 % vs 12 %, respectively [22]. Our finding is consistent with the chronology of early subcellular events following fertilization. At the earliest injection time point, both maternal and paternal genomes are not yet fully enclosed by their respective pronuclear membranes and therefore accessible. The male genome is also undergoing chromatin de-condensation, protamine removal and histone exchange [27–29]. Collectively, these events should facilitate transgene insertion. At the latest time point, when pronuclear membranes have fully formed and the pronuclei have reached their maximal size, access to the genome will be more restricted and the chances of integration reduced. Post-replicative transgene insertion into S-phase chromatin at this stage will result in mosaicism if only one of the two daughter cells inherits the transgenic chromosomes, whereas transgene insertion prior to DNA replication will decrease the likelihood of mosaicism. Taken together, we settled on injecting 20 ng/μL DNA at 7–8 hpi as optimized conditions for subsequent experiments.

### Cell counts of transgene-expressing blastocysts

We next characterized the morphological quality of blastocysts derived from our optimized injection conditions. There was no significant difference between randomly selected EGFP-positive and non-injected IVF blastocysts with respect to total cell counts (70 ± 10 *vs.* 74 ± 8, *P* > 0.05, Fig. 1b). This indicated that transgene-expressing blastocysts were not compromised in terms of overall morphological quality.

### Embryo transfer and detection of transgenic buffalo calves

**Fig. 2:**
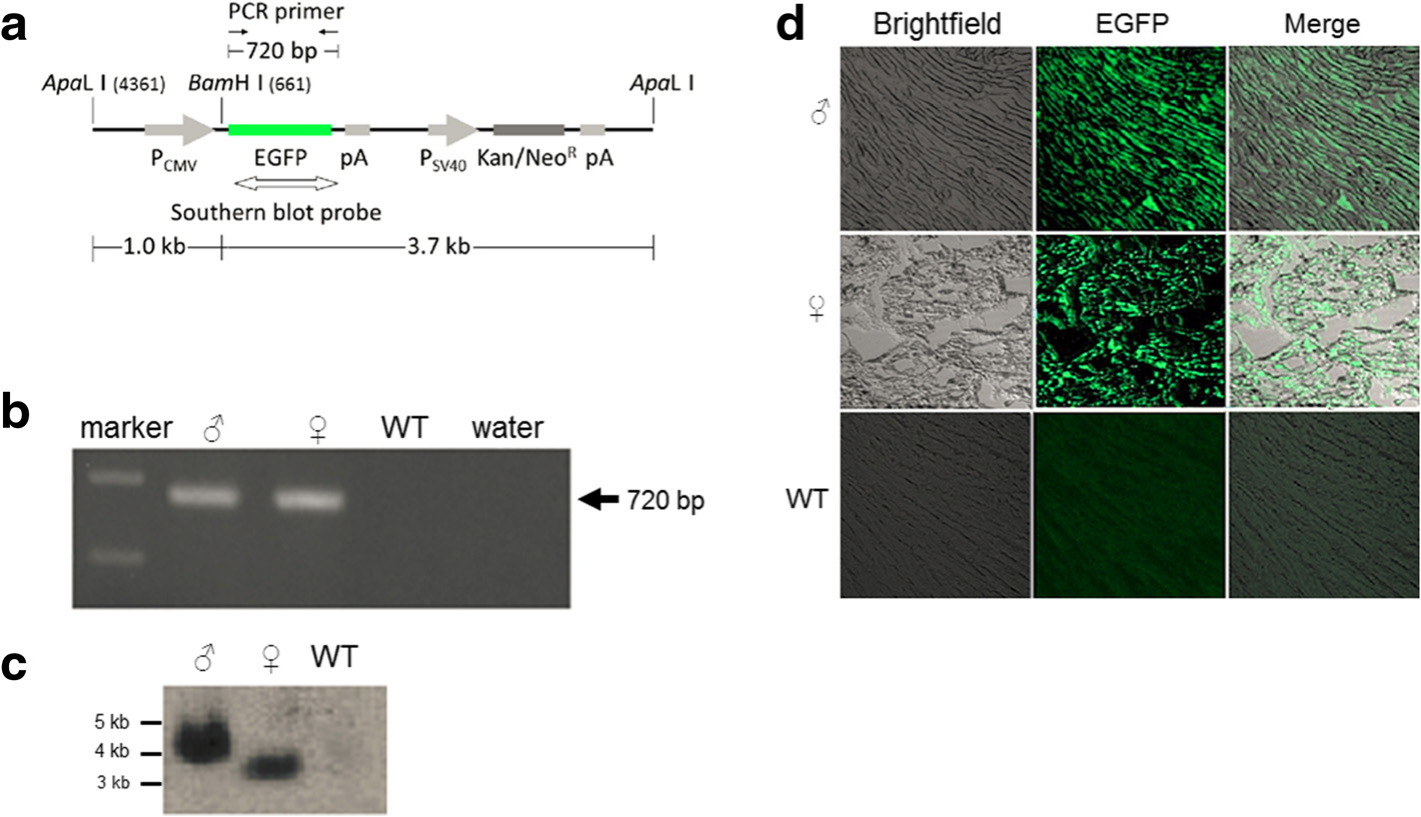
Characterization of transgenic buffalo calves derived from cytoplasmic zygote injection. **a**. Main features of injected pEGFP-N1 plasmid, showing restriction positions (in brackets), PCR primer binding sites (single arrows), Southern probe location (double block arrow) and expected amplicon sizes. p = promoter, pA = polyA site, R = resistance; **b**, **c**. Detection of EGFP by PCR (**b**) and Southern blot (**c**) in genomic DNA extracted from biopsied ear tissues of transgenic buffalo calves (male, female) and wild-type buffalo ear tissue (WT). Water provided a no template control. **d**. Ear tissue sections from transgenic calves (male, female) and wild-type control calf (WT) observed by confocal laser scanning microscopy (Brightfield, EGFP and merged images, respectively)

To determine their in vivo viability and capacity for full term development, six EGFP-positive blastocysts were transferred into the uterine horns of four cross-bred buffalo recipients (Table 3). One recipient was pregnant at D40 and two buffalo calves, one male and one female, were born on 2 December 2010 (Table 3, Fig. 1c). Since the GFP expression in embryos could reflect transient transcription of non-integrated DNA, both animals were analyzed for EGFP gene integration (Fig. 2a). Genomic DNA, extracted from ear biopsies of the two calves and a wild-type control calf, was used as a template. Both PCR and Southern blot specifically detected presence of the EGFP transgene in the two buffalo calves that developed from EGFP-positive blastocysts (Fig. 2b, c). For single-copy integration, the minimal size of the resulting *Bam*H I fragment recognized by the Southern probe would be 3.7 kb. For multi-copy integrations (head-to-head, head-to-tail or mixed), the next bigger fragment size would be 3.7 + 1.0 = 4.7 kb. Multi-copy insertion would also result in increased signal intensity. The observed fragment sizes and signal intensities are consistent with head-to-head multi-copy and single-copy integration in the male and female calf, respectively (Fig. 2c).

**Table 3:** Embryo transfer summary

Recipient	nET	Pregnancies at D40	Calves born
1	1	0	0
2	2	2	2
3	1	1	0
4	2	0	0

*nET* number of embryo transfers

We further used confocal laser scanning microscopy to directly observe EGFP expression in transgenic buffalo ear tissue cultures. EGFP signal was specifically observed in all cells from presumptive transgenic primary tissue cultures, indicating that the randomly integrated transgene was expressed and functional (Fig. 2d).

The male and female transgenic calves were born after twin embryo transfer. In cattle, the majority of females from mixed-sex dizygotic twin pregnancies are freemartins [30]. This condition is due to exchange of cellular material and hormones between the vasculary connected twin placentas. As a result, over 90 % of female twins have abnormally masculinized reproductive organs and are infertile. This phenomenon also occurs in buffalo [31]. In our case, the female transgenic calf was diagnosed as freemartin by ultrasound analysis, precluding it from subsequent mating. The male calf has so far not been mated and analyzed for transgene segregation in the offspring.

## Conclusions

We demonstrate that injecting 20 ng/μL exogenous DNA into buffalo zygotes at 7–8 hpi reproducibly results in >15 % transgene-expressing blastocysts. This net efficiency compares favorably to studies in mice and other livestock species, perhaps in part due to species-specific differences that affect stability and processing of exogenous DNA [32]. Using these optimized parameters, we produced the first transgenic buffalos from cytoplasmic injection. This technique would also be applicable to non-reporter transgenes, which could be detected on biopsies prior to embryo transfer [33, 34].

## Abbreviations

IVM: In vitro maturation
IVF: In vitro fertilization
hpi: Hours post-insemination
